# Evaluation of the validity of the pre-marriage mean corpuscular volume value as a predictive test for b-thalassemia carrier status

**DOI:** 10.5937/jomb0-37682

**Published:** 2023-03-15

**Authors:** Ali A. Almomani, Ala'a S. Shraim, Ali M. Atoom, Majeed Bayan A. Abdel, Jehad F. Alhmoud

**Affiliations:** 1 Al-Ahliyya Amman University, Pharmacological and Diagnostic Research Centre, Amman, Jordan; 2 Al-Ahliyya Amman University, Faculty of Allied Medical Sciences, Department of Medical Laboratory Sciences, Amman, Jordan

**Keywords:** premarital screening test, b-thalassemia minor, HPLC, HbA2, MCV, RDW, RBC count, predbračni skrining test, b-talasemija minor, HPLC, HbA2, MCV, RDV, broj eritrocita

## Abstract

**Background:**

The national mandatory premarital screening test is based on mean corpuscular volume (MCV) > 80 fL value for the detection of β-thalassemia to provide acceptance for marriage. The objective of this study is to assess the efficacy of MCV as a screening test for β-thalassemia trait in the present population.

**Methods:**

This study was conducted on 418 blood samples collected from adult individuals. The diagnosis of β-thalassemia carrier was given to those having HbA_2_ values equal to or above 3.5%. The diagnostic reliability of different RBC indices and formulas in discriminating cases of β-thalassemia trait were evaluated. Finally, a new index called "Momani" was determined based on MCV, RDW and RBC count.

**Results:**

β-thalassemia trait was identified in 10% of the cases. The measured MCV value was significantly lower in β-thalassemia carrier group compared to non-carrier group (p = <0.001). MCV value and RBC count showed a higher diagnostic reliability than other RBC indices. We found that MCV ≤ 74.45 fL is more suitable cutoff value of MCV with 86.2% specificity, 71.4% sensitivity, 36.6% positive predictive value, and 96.4% negative predictive value. Finally, our index "Momani" was found to be useful in predicting carrier and paralleled the performance of Sirdah, Mentzer, and Ehsani indices.

**Conclusions:**

MCV<80 is a useful but not a perfect cutoff point for the screening of β-thalassemia carriers from noncarriers. The diagnostic accuracy of MCV can be improved by selecting a new cutoff value. Moreover, "Momani" index shows good discrimination ability in diagnosing β-thalassemia carrier in our population.

## Abstract (sr)

**Uvod:**

Nacionalni obavezni predbračni skrining test je zasnovan na vrednosti srednjeg korpuskularnog volumena (MCV) > 80 fL za otkrivanje b-talasemije kako bi se obezbedilo prihvatanje braka. Cilj ove studije je da se proceni efikasnost MCV kao skrining testa za osobinu b-talasemije u sadašnjoj populaciji.

**Metode:**

Ova studija je sprovedena na 418 uzoraka krvi uzetih od odraslih osoba. Dijagnoza nosioca b-talasemije je data onima koji imaju HbA2 vrednosti jednake ili veće od 3,5%. Procenjena je dijagnostička pouzdanost različitih indeksa i formula eritrocita u diskriminatornim slučajevima osobine b-talasemije. Konačno, novi indeks pod nazivom "Momani" je određen na osnovu broja MCV, RDV i RBC.

**Rezultati:**

Osobina b-talasemije identifikovana je u 10% slučajeva. Izmerena vrednost MCV bila je značajno niža u grupi nosilaca b-talasemije u poređenju sa grupom koja nije nosila (p = <0,001). MCV vrednost i broj eritrocita pokazali su veću dijagnostičku pouzdanost od drugih indeksa eritrocita. Otkrili smo da je MCV ≤ 74,45 fL pogodnija granična vrednost MCV sa 86,2% specifičnosti, 71,4% osetljivosti, 36,6% pozitivne prediktivne vrednosti i 96,4% negativne prediktivne vrednosti. Konačno, pokazalo se da je naš indeks "Momani" koristan u predviđanju nosioca i da je uporedio performanse Sirdah, Mentzer i Ehsani indeksa.

**Zaključak:**

MCV<80 je korisna, ali ne i savršena granična tačka za skrining nosilaca b-talasemije od onih koji nisu nosioci. Dijagnostička tačnost MCV može se poboljšati izborom nove granične vrednosti. Štaviše, "Momani" indeks pokazuje dobru sposobnost diskriminacije u dijagnostici nosioca b-talasemije u našoj populaciji.

## Introduction

Hemoglobinopathies are defined as genetic disorders that causes an abnormal structure of the hemoglobin molecule. Usually, genetic mutations cause major alterations in the hemoglobin structure along with dysfunctions associated with changes in the hemoglobin shape, oxygen-carrying capacity, or ability for aggregation leading to blockage the vascular system [1]. As reported, hemoglobin disorders represent a significant health problem in 71% of 229 countries, which includes 89% of all births worldwide [2]. Recent surveys suggested that between 300,000 and 400,000 babies are born yearly with a serious hemoglobin disorder and approximately 90% of these births occur in low- or middle-income countries [3].

Thalassemia disease is the most common form of inherited anemia worldwide. WHO reported thatabout 1.5% of the world’s population might be carriers of thalassemia and about 60, 000 infants are born with a major thalassemia [4]. The distribution of thalassemia genes extends from the Mediterranean basin and Sub-Saharan Africa through the Middle East to the Far East, including South China and the Pacific Islands [5]. Moreover, β-thalassemia is encountered in almost all Arab countries with carrier rates ranging from 1 to 11% [6]. In Hashemite Kingdom of Jordan, β-thalassemia is one of the major inherited disorders with the carrier rate for the disease estimated to be 3–6% [7].

Despite the high rates of haemoglobinopathies, many Arab countries are facing major challenges in providing comprehensive and up-to-date care. Also, prevention services are crucial due to paucity of resources, presence of other competing priorities of disorders, such as cardiovascular diseases, cancer and diabetes, and the insufficient number of trained health care professionals in this field [6]. Nevertheless, genetic services are currently available in several Arab countries, such as newborn screening (NBS), premarital carrier screening (PMS), prenatal screening, genetic counselling, and education. The premarital screening program (PMS) is a comprehensive program that is mainly used by couples who are planning to get married in order to scan the presence of various inherited or acquired disorders [6].

In June 2004, the Jordanian Ministry of health has implemented a national mandatory premarital screening test for the detection of β-thalassemia and sickle-cell anemia [8]. The program undertakes screening by estimation of mean corpuscular volume (MCV) for both couples who contemplating marriage. When the MCV levels are less than 80 fL for both couples, their blood samples are further analyzed by hemoglobin electrophoresis to estimate the hemoglobin A_2_ (HbA_2_) level. The HbA_2_ values equal to or more than 3.5% are considered β-thalassemia carriers [7]. Hemoglobin A_2_ estimation is the gold standard for the diagnosis of β-thalassemia trait [9]. A published study has correlated HPLC (High performance liquid chromatography) in the characterization of hemoglobin profile in thalassemia syndromes with the hemoglobinopathies and proved that HPLC was an outstanding powerful diagnostic device for the identification of hemoglobin variants with a high precision degree in the quantification of normal and abnormal hemoglobin fractions [10]. In addition, the sensitivity and specificity of HPLC was found to be 100% and 99.99%, respectively [11]. However, until now, the Ministry of health depends only on MCV>80 fL value to provide acceptance of the marriage, without being further tested by electrophoresis method. Additionally, the Jordanian individuals are not adhering to the obligatory law by the Ministry of health to perform this test because of the high cost of HPLC method. As a result, a small percentage of β-thalassemia minor escapes to be diagnosed and that may be the source emergence of new emerging of β-thalassemia major cases. Therefore, as no effective treatment exists for β-thalassemia and affected individuals require regular blood transfusion, this can represent a major financial and psychological load on the patients and their families and adds further challenges to the national healthcare system [12].

The main aim in this study is to assess the efficacy of the national premarital screening test which is based on the MCV parameter to detect the undiagnosed thalassemia trait cases. Moreover, the pattern of hemoglobin electrophoresis in our study will be detected using the HPLC method. Then, the HPLC results will be statistically compared with the MCV parameter to justify whether this RBC parameter is enough to be a confirmatory test for pre-marriage or not. The study hypothesized that undiagnosed cases of β-thalassemia trait play a critical role in the prevalence of the disease and specifically could be the result of emerging new β-thalassemia major cases.

## Materials and methods

### Complete blood count (CBC)

CBC was analyzed using BC-5300 automated hematology analyzer from Mindray (Shenzhen Mindray Bio-Medical Electronics, China) that provided an automatic measurement of 20 parameters which include: RBC Count, Hemoglobin, Hematocrit, Mean Corpuscular Volume (MCV), Mean Corpuscular Hemoglobin (MCH), Mean Corpuscular Hemoglobin Concentration (MCHC), and Red Cell Distribution Width (RDW-CV, RDW-SD). The principle of the device was highlighted by several methods which are: Electrical impedance method for counting cellular elements of blood; Laser light scatter for distinguishing between granulocytes, lymphocytes, and monocytes; Cellular fLuorescence to measure RNA (reticulocytes), DNA (nucleated red cells), and cell surface antigens; Light absorption for measuring the concentration of hemoglobin; and Electrical conductivity to determine physical and chemical composition of leucocytes for their classification.

### High Performance Liquid Chromatography (HPLC)

HbA_2_ level was measured using Automated Glycohemoglobin Analyzer HLC-723 from TOSOH Corporation (Tokyo, Japan). This device provides an instruction manual that shows hemoglobin electrophoresis in which the area under the peak is measured subsequently. The assay principle depends on the separation achieved by utilizing differences in the ionic interactions between the cation exchange group on the resin surface and the hemoglobin components. HbF and HbA_2_ can be measured by performing a stepwise elution using the TSK gel G7 β-thalassemia and three elution buffers with different salt concentrations and pH.

### Study participants and ethical consideration

The blood samples (418) included in our study were collected from Al Jama’a Medical Laboratory located in Irbid, Jordan between April 2021 to July 2021. Patients’ blood samples were tested for CBC and Hemoglobin electrophoresis by HPLC and comprised of 112 males and 306 females in the age of marriage (22–45 years old). All participants were acknowledged about the aim of the study and the procedure, subsequently, consent form written in Arabic was signed by each participant prior to sample collection. Furthermore, all data collected for the study were handled with strict confidentiality according to Helsinki ethical guidelines. All participants were asked to fill out a medical history questionnaire written in Arabic that included personal information (name, age, national ID, gender, contact number) and medical history information on whether the participant suffers from chronic diseases such as iron deficiency anemia, B_12_ deficiency anemia, autoimmune deficiency, diabetes mellitus, coronary disease, and leukemia. This study was approved by the Ethical Review Committee of the Faculty of Allied Medical Sciences at Al-Ahliyya Amman University with the approval number (IRB; AA-3-3-21).

### Sample collection and processing

5 mL blood sample was withdrawn from each participant in an EDTA anticoagulated tube and hence, this sample was used for both CBC and HbA_2_ level measurement. Then, blood samples werechecked for any clot formation; hence, any sample revealed a clot was rejected and excluded from our study. HbA_2_ level was detected by high-performance liquid chromatography (HPLC) method using TOSOH device. Finally, CBC measurement was determined using Mindray BC-5000 analyzer.

### Statistical analysis

Continuous variables were summarized with the mean, standard deviation, and range. The normality of data distributions was checked with Kolmogorov-Smirnov and Shapiro-Wilk tests. Due to lack of normality, the differences between variables were tested using the nonparametric Mann-Whitney U test. Values < 0.05 were considered statistically significant. The diagnostic ability of indices was illustrated using the receiver operating characteristic (ROC) curve, which is used to calculate the area under the curve (AUC) for each index and formula. The ROC was also used to choose the optimal cut-off value which maximizes the sensitivity and specificity. The diagnostic accuracy of an index and its cut-off were assessed through a confusion matrix of true and false positives and false negatives when compared to the HbA_2_ gold standard. Sensitivity, specificity, negative as well as positive predictive power and Youden’s index were calculated from the frequencies of true and false positives and negatives according to the formulas given below:

Sensitivity = TP/ (TP + FN), Specificity = TN/ (TN + FP), Positive predictive value = TP/ (TP +FP), Negative predictive value = TN/ (TN + FN), Accuracy = True positive + true negative / (true positive + true negative + false positive + false negative), Youden’s index = sensitivity + specificity − 1. The 11 discriminant indices used in the evaluation are summarized in Table 1. Linear discriminant analysis was used to find a new diagnostic index composed of a linear combination of blood parameters that separates β-thalassemia carriers and noncarriers. All calculations and figures were produced using SPSS version 22.0 (IBM Corp. Released 2013. IBM SPSS Statistics for Windows, Version 22.0. Armonk, NY: IBM Corp).

**Table 1:** Diagnostic accuracy measuring of blood parameters in diagnosing β-thalassemia carrier status (n=418). PPV: Positive predictive value, NPV: Negative predictive

Parameter	Cut-off	Sensitivity	Specificity	Accuracy	PPV	NPV	Youden’s index
MCV	< 80 fL	71.4	71.0	71.1	21.6	95.7	42.4
MCH	< 1.67 fmol	69.0	69.4	69.3	20.1	95.3	38.4
MCHC	< 20.7 mmol/L	66.7	63.6	63.9	17.0	94.5	30.3
RDW	> 13.95%	66.7	65.4	65.6	17.7	94.6	32.1
RBC	> 4.995	73.8	69.9	70.3	21.5	96.0	43.7

## Results

### Descriptive statistics for blood parameters

In our study, The CBC and HbA_2_ data of 418 participants (306 females and 112 males) are summarized in Table 2. Females had an average MCV of 80.7 fL (±9.1), whereas males had an average of 82.3 fL (±9.2). Moreover, females had an average HbA_2_ percentage of 2.6% (±1.1) compared to 3.1% (±1.6) for males. The differences in all the parameters between males and females were significant p <0.05 (Mann-Whitney U test).

**Table 2:** Descriptive statistics for blood parameters of the study sample categorized according to gender. Females (n=306), Males (n=112). P value < 0.05: significance level from Mann-Whitney U test.

Parameter	Gender	Statistic		MWU<br>(p)	Range	
		Mean	SD		Minimum	Maximum
MCV (fL)	Female	80.7	9.1	0.04	53.9	109.8
	Male	82.3	9.2		56.7	98.2
MCH (fmol)	Female	1.67	0.2	0.001	0.99	2.18
	Male	1.76	0.2		1.12	3.66
MCHC (mmol/L)	Female	20.6	0.8	<0.001	17.2	23.0
	Male	21.1	0.8		17.9	22.6
RDW - CV (%)	Female	14.3	2.1	<0.001	11.8	24.2
	Male	13.5	1.4		11.8	19.6
Hb concentration<br>(g/L)	Female	121	17	<0.001	68	167
	Male	154	14		105	183
Hematocrit (L/L)	Female	0.36	0.04	<0.001	0.19	0.46
	Male	0.45	0.03		0.33	0.53
RBC count (10^12^/L)	Female	4.5	.57	<0.001	2.0	6.8
	Male	5.6	.64		4.0	7.7
HbA_2_ (%)	Female	2.6	1.1	<0.001	.3	8.3
	Male	3.1	1.6		1.1	9.6

### Descriptive statistics for blood parameters based on β-thalassemia carrier status

Forty-two individuals (10.0% of the whole sample) were detected as β-thalassemia carriers based on HbA_2_ percentage equal to or above 3.5% which was measured by HPLC method. As shown in Table 3, the average HbA_2_ percentage of carriers was 5.9% (±1.7), and 2.4% (±0.53) for non-carriers. Carriers had an average MCV of 69.5 fL (±11.3) as compared to 82.5 fL (±7.9) for non-carriers (see Table 3). The difference in MCV was statistically significant as indicated by Mann Whitney U test. In addition, mean corpuscular hemoglobin (MCH) and mean corpuscular hemoglobin concentration (MCHC) were significantly lower in carriers than in non-carriers. On the other hand, red cell distribution width (RDW) and RBCs count were significantly higher in carriers than in noncarriers. Only hemoglobin concentration (Hb) and hematocrit (Hct) were not found to be significantly different between carriers and non-carriers. This indicates that hemoglobin concentration and hematocrit may not be useful in distinguishing between carriers and non-carriers β-thalassemia cases.

**Table 3:** Descriptive statistics for blood parameters of the study sample categorized according to β-thalassemia carrier status. Carriers (n=42), Non-carriers (n=376). P value < 0.05: significance level from Mann-Whitney U test.

Parameter	Carrier status based on HbA_2_ ≥ 3.5%				
	Carrier (n=42)		Non carrier (n=376)		MWU
	Mean	SD	Mean	SD	(p)
MCV (fL)	69.5	11.3	82.5	7.9	<0.001
MCH (fmol)	1.48	0.4	1.72	0.2	<0.001
MCHC (mmol/L)	20.5	0.8	20.8	0.8	0.002
RDW - CV (%)	14.7	1.7	14.1	2.0	0.001
Hb Concentration (g/L)	128	18	130	22	0.470
Hematocrit (L/L)	0.38	0.04	0.38	0.06	0.918
RBC count (10^12^/L)	5.7	0.95	4.7	0.65	<0.001
HbA_2_ (%)	5.9	1.7	2.4	0.53	<0.001

In the same context, male and female β-thalassemia carriers did not differ significantly in their MCV, MCH, MCHC, and RDW as shown in Table 2. They were also not significantly differed in their HbA_2_ percent values (p=0.271). Yet, male, and female carriers were significantly different in terms of hemoglobin concentration (Hb), hematocrit (Hct), and RBC count. This indicates that gender may not be an important factor in identifying β-thalassemia carriers since most blood parameters that were found to be different between carriers and non-carriers are not significantly different between male and female carriers (see Table 2). However, male and female were statistically significantly different regarding all blood parameter values (see Table 2).

### Performance of raw blood parameters in predicting β-thalassemia carrier status

**Figure 1:**
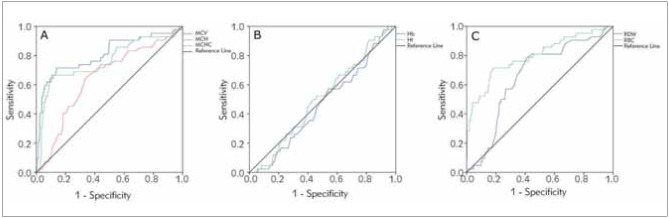
Receiver operating characteristic (ROC) curves illustrating the diagnostic abilities of raw blood parameters in recognizing β-thalassemia carriers compared to HbA_2_ gold standard. a. ROCs for the blood parameters: MCV, MCH, and MCHC that were lower in carriers than in non-carriers (see Table 3). b. ROCs for the blood parameters that were not different in carriers and in non-carriers: Hb and Hct. c. ROCs for the blood parameters that were higher in carriers than in non-carriers: RDW and RBC.

Our data indicated that all raw blood parameters were able individually to distinguish between β-thalassemia carriers and non-carriers except for Hb concentration and Hct (see Figure 1). This is expected since Hb and Hct were not significantly different between carriers and non-carriers as shown previously in Table 3.

Moreover, our data demonstrated that MCV was the best blood parameter in distinguishing between carriers and non-carriers with an area under (AUC) the ROC curve of 0.807. MCV was followed by RBC count with an AUC of 0.795 and MCH with an AUC of 0.760 (see Table 3). All parameters had AUCs significantly greater than 0.5 (a non-informative diagnostic test) except for Hb and Hct, which had p values > 0.05 indicating that the diagnostic abilities of these two parameters are not different from assigning carrier status at random.

The diagnostic accuracy measures for raw blood parameters in discriminating between carriers and non-carriers are detailed in Table 1. Hb concentration and Hct were excluded from this table since they were found to be uninformative regarding β-thalassemia carrier status. Using a cutoff value of 80 fL for MCV that is used in the Jordanian premarital screening test for β-thalassemia carrier status, the sensitivity and specificity were about 71%. The MCH with a cutoff value 27 pg (based on literature) and RBC with a cutoff value of 4.995 (based on maximum Youden’s index from ROC) had similar sensitivity and specificity to that of MCV. The maximum Youden’s index was calculated for MCHC, RDW and RBC from their respective ROC curves, and the corresponding cutoff value was used for accuracy measure calculations as shown in Table 1. As demonstrated, all parameters had Youden’s indices less than 50, which means that none of them is particularly efficient in balancing sensitivity and specificity.

In addition, it is well known that prevalence affects both the positive and negative predictive value of a diagnostic test. As our data shown, the prevalence of β-thalassemia trait in our sample is 10%. Accordingly, as prevalence decreases, the positive predictive value decreases, and the negative predictive value increases. This might explain the low positive predictive value and high negative predictive value of all parameters as indicated in Table 1.

### Performance of discriminant indices in predicting β-thalassemia carrier status

**Figure 2:**
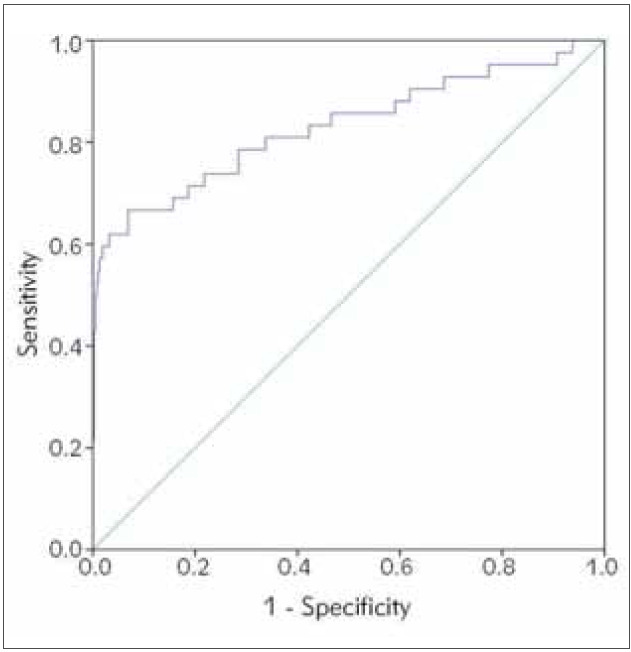
Receiver operating characteristic (ROC) curve illustrating the diagnostic ability of a »Momani« index based on MCV, RDW and RBC count in recognizing β-thalassemia carriers compared to HbA_2_ gold standard.

Eleven discriminant indices with their formulas and cut-off values were used for the prediction of β-thalassemia carrier status in our present samples (Table 1). The calculated means (±SD) and ranges for these indices are listed in Table 4. In addition, all indices were significantly different between β-thalassemia carriers and non-carriers (Mann-Whitney U p-value < 0.05) as demonstrated in Table 5. This indicates that all these indices are potentially useful in distinguishing between carriers and non-carrier cases. Moreover, all blood formulas were capable of distinguishing carriers from non-carriers except for Ricerca and MCHD indices (Figure 2). These two indices reversed the diagnosis misclassifying carriers as noncarriers and vice versa.

**Table 4:** Descriptive statistics for the study sample (n=418) categorized according to carrier status based on HbA_2_ gold standard and MCV< 80 fL. P value: Significance level from Mann-Whitney U test.

Parameter	Carrier status based on HbA_2_ ≥ 3.5%									
	Carrier (n=42)					Not carrier (n=376)				
	Normal MCV<br>(n=12)		MCV<80<br>(n=30)		MWU<br>(p)	Normal MCV<br>(n=267)		MCV <80<br>(n=109)		MWU<br>(p)
	Mean	SD	Mean	SD		Mean	SD	Mean	SD	
MCV	85.5	3.9	63.0	5.2	<0.001	86.5	3.9	72.6	6.4	<0.001
MCH	1.82	0.07	1.35	0.45	<0.001	1.83	0.09	1.47	0.18	<0.001
MCHC	21.3	0.44	20.1	0.62	<0.001	21.1	0.60	20.1	0.86	<0.001
RDW	13.3	0.81	15.3	1.6	<0.001	13.4	1.1	15.7	2.6	<0.001
Hb	139	17	123	16	<0.001	136	19	115	23	0.01
Hct	0.40	0.04	0.37	0.04	<0.001	0.39	0.05	0.35	0.06	0.138
RBC	4.8	0.54	6.0	0.82	0.006	4.6	0.63	4.9	0.60	<0.001

**Table 5:** The frequencies of true carriers and non-carriers within the carrier status based on MCV≤ 74.45 fL.

Carrier state based on<br>MCV ≤ 74.45 fL	True carrier state based<br>on HbA_2_ ≥ 3.5%	Frequency	Percent	Status
Not a carrier	Not a carrier	324	96.4	True negative
	Carrier	12	3.6	False negative
	**Total**	**336**	**100.0**	
Carrier	Not a carrier	52	63.4	False positive
	Carrier	30	36.6	True positive
	**Total**	**82**	**100.0**	

As demonstrated in Table 6, Sirdah, Mentzer, Ehsani and RDW indices were the best performing diagnostic tests for β-thalassemia carrier status based on our data with AUC under ROC curves equal 0.830, 0.827, 0.825, and 0.820 respectively (see Table 6). Areas under ROC curves were significantly different (p value <0.001) from 0.5 for all indices. However, Ricerca and MCHD had AUCs less than 0.5 indicating a reversal of diagnosis based on the published cutoff value when was applied to our study sample. The diagnostic accuracy measures of these indices in discriminating between carriers and noncarriers are detailed in Table 7. As shown, Green and King, MDHL, and RDW indices balanced sensitivity and specificity better than the rest. Even though Mentzer, Sirdah, and Ehsani had some of the best AUCs under the ROC, the published cutoffs had low sensitivity when applied to our data, which indicated that these cutoffs were unable to recognize carriers efficiently. Ricerca and MCHD had very poor sensitivity because of reversing classification labels as indicated in Figure 2: B.

**Table 6:** Diagnostic accuracy measuring of our new index in diagnosing β-thalassemia carrier status PPV: Positive predictive value, NPV: Negative predictive value.

Parameter	Cutoff	Sensitivity	Specificity	Accuracy	PPV	NPV	Youden’s index
Momani index	≤ -0.2394	78.6	71.5	72.3	23.6	96.8	50.1

**Table 7:** Areas under the receiver operating curves of the best performing indices for the diagnosis of β-thalassemia carrier status. P value: Significance level of difference from an area of 0.5 (a non-informative diagnostic test).

Index	Area under the receiver<br>operating curve (AUC<br>under ROC)	P value	95% Confidence Interval for area under the curve	
			Lower Bound	Upper Bound
Sirdah	0.830	< 0.001	0.749	0.911
Momani	0.830	< 0.001	0.747	0.913
Mentzer	0.827	< 0.001	0.746	0.909
Ehsani	0.825	< 0.001	0.742	0.908
RDWI	0.820	< 0.001	0.739	0.901
Green and King	0.815	< 0.001	0.733	0.896

### Validity of MCV < 80 fL in predicting β-thalassemia carrier state

Twelve true carriers (28.6%) as determined by HbA_2_ gold standard had normal MCV values larger than 80 fL. Moreover, 109 true non-carriers (29.0%) had MCV values less than 80 fL (Table 4). This indicates that MCV< 80 is not a perfect diagnostic test to distinguish carriers from non-carriers. The twelve true carriers who had MCV > 80 fL were seven females and five males. However, MCV is still a useful screening test since the mean of MCV values in carriers is significantly lower than in non-carriers (see Table 3). Specifically, mean MCV in carriers equal 69.5±11.3 fL, and in non-carriers equal 82.5±7.9 fL with p-value <0.001.

In addition, the carrier group based on MCV<80 has a higher percentage of true carriers (21.6% vs. 4.3%) and a lower percentage (78.4% vs. 95.7%) of true non-carriers than the group judged as non-carrier (Figure 3). This difference between MCV carriers and non-carriers in proportions of true carriers and non-carriers are statistically significant (Pearson’s Chi-squared test: x^2^ = 28.775, df = 1, p-value = 8.131x10^-8^). As indicated in Table 3, MCV <80 fL had a sensitivity (ability to identify thalassemia trait) of 71.4% and specificity (ability to recognize the absence of thalassemia trait) of 71.0%, and a Youden’s index of 42.4.

In an attempt to improve the performance of MCV in predicting the carrier state, the maximum Youden’s index from the MCV ROC curve was used to determine a new cutoff value of 74.45 fL instead of <80 fL. The performance of this new cutoff value is shown in Table 5. The diagnostic accuracy measures for the new suggested cutoff MCV value have been determined and shown a better diagnostic accuracy than the original 80 fL cutoff with a specificity of 86.2% (versus 71.0%), a sensitivity of 71.4% (same), a positive predictive value of 36.6% (versus 21.6%), a negative predictive value of 96.4% (versus 95.7%) and a Youden’s index of 57.6 (versus 42.2). Thus, MCV is a valid test for diagnosing the carrier status, but it can be improved by selecting a better threshold.

By using discriminant analysis, a new index was found to be useful in predicting carrier status based on MCV, RDW and RBC count. Momani index = -7.665 +(0.106×MCV) +(0.164×RDW) -(0.679×RBC)

The ROC of this index is shown in Figure 2. The AUC of the ROC is 0.830 (95% CI: 0.747–0.913), and this area was significantly different from an area of 0.5 (p <0.001). Based on the maximum Youden’s index, a cutoff value of ≤ -0.2394 was determined for the new index. The diagnostic accuracy of this cutoff is shown in Table 6. The best performing indices can be judged based on the area under the ROC curve. The larger the AUC, the better the diagnostic ability of the index. Therefore, according to our data the best performing indices are ordered in Table 7. Our new »Momani« index paralleled the performance of Sirdah, Mentzer, and Ehsani indices.

## Discussion

HbA_2_ is a type of hemoglobin, which plays a critical role in β-thalassemia trait screening. Noteworthy, studies have proved that the level of HbA_2_ increases in β-thalassemia trait carriers while reduces sometimes, in cases with IDA and α-thalassemia trait [13]. The normal range of HbA_2_ level is 2.4%–3.2%, while in β-thalassemia trait the HbA_2_ level ranges between 3.6% and 7% [13]. A high HbA_2_ level above or equal to 3.5% with reduced MCV and MCH values are widely reported as a typical diagnosis and valuable parameters for β-thalassemia detection [14]. Additionally, β-thalassemia trait causes a decrease Hb concentration and Hct due to insufficient viable cells in the blood stream as RBC precursor suffer from premature death in the bone marrow besides the presence of inclusion bodies which lead to destroying RBCs by activated macrophages [15]. In agreement with these studies, our data demonstrated that the average level of HbA_2_ in the detected β-thalassemia carriers was 5.9% whereas, the average level of HbA_2_ in non-carrier cases was 2.4%. Hb concentration and Hct were significantly decreased in β-thalassemia carriers compared to non-carriers. This finding is consistent with a previous study, which demonstrated that carriers of β-thalassemia trait may have reduced Hb with mild anemia [16]. On the other hands, our data indicated that the RBCs count are significantly induced in β-thalassemia carriers, which presumably as a result of promptly increases the production of RBCs by the bone marrow in β-thalassemia. Nevertheless, these cells are insufficient viable calls and rapidly destroyed by macrophages since they are sequestered in spleen (extravascular hemolysis), in addition to the existence of target cells, elliptocytes, and basophilic stippling which resulting in an increase of RBC destruction and ineffective erythropoiesis at later stages. All together induce bone marrow hyperplasia [15] and hence high RBCs count as clearly demonstrated in our data shown in Table 3. In the same context, RDW-CV was significantly higher in β-thalassemia carries relative to non-carries which is more probably correlated with the degree of anisopoikilocytosis [10]. In addition, the results of our study also demonstrated that all measured blood parameters (MCV, MCH, MCHC, RDW-CV and RBC count) are significantly different between β-thalassemia carrier and non-carrier cases. On the other hands, Hb concentration and Hct may not be useful RBC indices in distinguishing between carrier and non-carries cases.

Moreover, out of 418 subjects recruited in our study, 42 cases (10%) are considered β-thalassemia trait carriers which includes 23 females and 19 males with no significant differences in MCV, MCH, MCHC, and RDW values between them. Thus, gender may not be considered a crucial factor in identifying β-thalassemia carriers. It is worth mentioning here that β-thalassemia is directly affecting the shape and size of the RBCs and may cause anisopoikilocytosis which can be recognized by RBCs indices in particular RDW index and this may lead to minimize the differences of these parameters between genders in the carrier group [17]. However, in non-carrier group all RBCs parameters were found to be significant among males and females, this may be due to increased physiological demands and decreased iron stores in females especially during the menstrual cycle. High muscles mass and skeletal structure in males induce RBCs production and cell compensation by the bone marrow [18].

The diagnostic ability of the RBC indices was illustrated using the receiver operating characteristic (ROC) curve. The findings showed that all parameters were significantly different in the β-thalassemia carrier group compared to non-carrier group. Thus, RBC parameters can distinguish between β-thalassemia carriers and non-carriers except for Hb concentration and Hct which were not significant. As indicated MCV was the best blood parameter in distinguishing between carriers and non-carriers with an area under (AUC) the ROC curve of 0.807. MCV was followed by RBC count with an AUC of 0.795 and MCH with an AUC of 0.760. This finding is in good agreement with the previously reported data by Baliyan et al. [11], 2019 who stated that MCV had a better AUC (0.650) than MCH (0.635). Hence, measuring the RBC indices could be a good parameter for screening methods for β-thalassemia identification.

In the light of these data, the diagnostic accuracy of RBCs indices in discriminating between β-thalassemia carriers and non-carriers were assessed when compared to the HbA_2_ gold standard as indicated in Table 1. Hb concentration and Hct were excluded from this table since they were found to be uninformative regarding β-thalassemia carrier status. As clarified MCV index with cut off value 80 fL showed the highest diagnostic accuracy among all tested RBC indices. The calculated sensitivity and specificity were 71.4% and 71%, respectively. Followed by RBCs count with a cutoff value of 4.995 (10^12^/L) which had similar sensitivity and specificity to that of MCV. Then, MCH index with a cutoff value 1.67 fmol (27 pg) which showed sensitivity and specificity about 69.0 %. Mendiratta, et al. [9], did a comparison study for the screening of β-thalassemia carrier status in one thousand women in the antenatal period. Here, the researcher found that the specificity of MCV (< 80 fL), RBC count (>5 million/mL^3^), and MCH (< 27 pg), was 85.88%, 93.8% and 74.16%, respectively. In addition, the sensitivity of MCV, RBC count, and MCH was 73.42%, 32.9%, and 60.76%, respectively [9]. Our result also showed that MCH is not more accurate than MCV in discriminating individuals with β-thalassemia trait. This finding contrasts with Karimi & Rasekhi [19] study which demonstrated that MCH (< 27 pg) had a sensitivity about 1% more than that of MCV (<80 fL). Since 1973, several distinguishing indications have been proposed for the quick and low-cost diagnosis of β-thalassemia trait and for the distinction between β-thalassemia trait and iron deficiency anemia [20] [21] [22] [23] [24] [25] [26] [27] [28] [17] [29] [30] [31] [32] [33] [34] [35]. Of these, eleven different RBC indices and mathematical formulas were used in our study to differentiate between β-thalassemia carriers and non-carriers. These formulas are summarized in Table 1. Combined RBC indices were included in the formulation of these equations which play a role in improving their predictive performance much better than when using one RBC index individually for example using MCV index alone.

The diagnostic accuracy of these previously published formulas was evaluated. The RBC parameters were applied to each formula, and a ROC curve was plotted for each one to calculate the area under the curve (AUC), and to assess its discriminative effectiveness in β-thalassemia detection. The formula will be more accurate and reliable when the area under the ROC curve is greater. The AUC of 1.0 represents the best differentiation while AUC of 0.5 represents the least valuable one. Our data revealed that none of the studied indices and formulas demonstrated 100% precision. Sirdah [26], Mentzer [20], and Ehsani, et al. [27] and RDWI indices provided the highest reliabilities for predicting β-thalassemia carrier status in our population sample (n=418) with AUC under ROC of 0.830, 0.827, 0.825, and 0.820 respectively. However, Ricerca (RDW/RBC) and MCHD (MCH/MCV) were poor and ineffective because they had AUCs less than 0.5 indicating a reversal of diagnosis.

Previous studies reported different sensitivities and specificities for the formulas we evaluated in this study which can be attributed to the differences in the distribution of hemoglobin b-subunit mutations, since each population has its specific distribution which is more likely may affect RBC indices and so the performance of these mathematical formulas [36] [37].

The main aim of this study is to evaluate the validity of using MCV (<80 fL) in the premarital screening test for predicting β-thalassemia carrier state. The data in Table 4 apparently revealed that out of 418 participants, 12 individuals (28.6%) out of 42 β-thalassemia carriers as determined by HbA_2_ gold standard had MCV values above 80 fL, while 109 cases (28.9%) out of 376 non-carriers had MCV less than 80 fL. These results provide clear evidence that using of MCV index alone may mask true carrier patients. Our data are in excellent agreement with several studies which demonstrated that using MCV alone for the screening of β thalassemia may pose a significant number of false negative and false positive results [11] [38]. However, MCV is still a useful screening test since the mean of MCV values in carriers is significantly lower than in non-carriers. Specifically, mean MCV in carriers equals 69.5±11.3, and in noncarriers equals 82.5±7.9 with p-value <0.001. In addition, the carrier group based on MCV <80 has a higher percentage of true carriers (21.6% vs. 4.3%) and a lower percentage (78.4% vs. 95.7%) of true non-carriers than the group judged as non-carrier. By using Youden’s index, we suggest a new cut-off value of MCV 74.45 fL which may improve the ability of the MCV index for predicting carrier status. Our data certainly clarified that the diagnostic performance of MCV index was improved where the percentage of true positive cases in carrier group was improved (36.6 %) compared with (21.6 %) using the ordinary cut-off value of MCV<80 fL. The diagnostic accuracy measures for the new suggested cutoff MCV value have been also determined and shown a better diagnostic accuracy than the original 80 fL cutoff with a specificity of 86.2% (versus 71.0%), a sensitivity of 71.4% (same), a positive predictive value of 36.6% (versus 21.6%), a negative predictive value of 96.4% (versus 95.7%), and a Youden’s index of 57.6 (versus 42.2). Thus, MCV is a valid test for diagnosing the carrier status, but it can be improved by selecting a better threshold. This finding is consistent with previous study which proved that 72 fL is the most suitable cutoff for MCV for diagnosing β-thalassemia trait with improved specificity and sensitivity [11].

A practical formula with high sensitivity and specificity is essential to discriminate between β-thalassemia trait and other causes of microcytic hypochromic anemia, specifically in premarital counseling and screening. Hence, it is important for physicians of different populations to establish their own formulas or evaluate the accuracy of published formulas for their own population to discriminate β-thalassemia trait from other causes of microcytic hypochromic anemia [37]. Therefore, we proposed a new index, which was proved to be more useful in predicting β-thalassemia carrier status based on MCV, RDW, and RBC count. The diagnostic accuracy measures of our proposed equation were calculated and compared with the previous published formulas. The AUC of this index is 0.83 which is equivalent to the performance of Sirdah [26], Mentzer [20], and Ehsani [27] et al. indices.

## Conclusion

Our study demonstrated that MCV<80 fL is useful cutoff value for the diagnosis of β-thalassemia trait carries. However, the new calculated cutoff value is very helpful in estimating the risk of minor β-thalassemia, particularly in cases with low MCV values. In addition, even though none of the indices or formulas provided 100% sensitivity or specificity for discriminating purposes, in a limited resource area such as our country, our proposed cutoff value of MCV and index may have great value in predicting β-thalassemia carrier status in Jordanian population.

## Dodatak

### Conflict of interest statement

All the authors declare that they have no conflict of interest in this work.

## References

[1] Keber B, Lam L, Mumford J, Flanagan B (2019). Hematologic Conditions: Common Hemoglobinopathies. FP essentials.

[2] Modell B, Darlison M J (2008). Global epidemiology of haemoglobin disorders and derived service indicators. Bull World Health Organ.

[3] de Sanctis V, Kattamis C, Canatan D, Soliman A T, Elsedfy H, Karimi M (2017). β-thalassemia distribution in the old world: an ancient disease seen from a historical standpoint. Mediterr J Hematol Infect Dis.

[4] Aydinok Y J H (2012). Thalassemia. Hematology.

[5] Angastiniotis M, Lobitz S J (2019). Thalassemias: An Overview. Int J Neonatal Screen.

[6] Hamamy H A, Al-Allawi N A (2013). Epidemiological profile of common haemoglobinopathies in Arab countries. J Community Genet.

[7] Hamamy H, Al-Hait S, Alwan A, Ajlouni K (2007). Jordan: Communities and community genetics. Community Genet.

[8] 8. Higher health council The National Strategy for Health Sector in Jordan 2015-2019. https://www.fpfinancingroadmap.org/resources/jordannational-health-sector-strategy-2015-2019.

[9] Mendiratta S, Bajaj S, Popli S, Singh S (2015). Screening of women in the antenatal period for thalassemia carrier status: Comparison of NESTROFT, red cell indices, and HPLC analysis. J Fetal Med.

[10] Khera R, Singh T, Khuana N, Gupta N, Dubey A J (2015). HPLC in characterization of hemoglobin profile in thalassemia syndromes and hemoglobinopathies: A clinicohematological correlation. Indian J Hematol Blood Transfus.

[11] Baliyan M, Kumar M, Nangia A, Parakh N J (2019). Can RBC indices be used as screening test for beta-thalassemia in Indian antenatal women?. J Obstet Gynaecol India.

[12] Hinda D, Qubbaj W, Abu R Z, Shboul M, Natasha S, El-Khateeb M β-Thalassemia: Prevention in Jordan. https://www.ncd.org.jo/docs/genetics/B-Thalassemia.pdf.

[13] Uçucu S, Karabıyık T, Azik F (2022). Teškoće u dijagnozi HbS-beta talasemije - zaista blaga bolest?. J Med Biochem.

[14] Barrett A N, Saminathan R, Choolani M (2017). Thalassaemia screening and confirmation of carriers in parents. Best Pract Res Clin Obstet Gynaecol.

[15] Keohane E M, Otto C N, Walenga J M (2019). Rodak's Hematology-E-Book: Clinical Principles and Applications.

[16] Galanello R, Origa R (2010). Beta-thalassemia. Orphanet J Rare Dis.

[17] Jayabose S, Giamelli J, Levondoglu-Tugal O, Sandoval C, Ozkaynak F, Visintainer P (1999). Differentiating iron deficiency anemia from thalassemia minor by using an RDW-based index. J Pediatr Hematol Oncol.

[18] Nah E H, Kim S, Cho S, Cho H (2018). Complete Blood Count Reference Intervals and Patterns of Changes Across Pediatric, Adult, and Geriatric Ages in Korea. Ann Lab Med.

[19] Karimi M, Rasekhi A J (2002). Efficiency of premarital screening of beta-thalassemia trait using MCH rather than MCV in the population of Fars Province, Iran. Haematologia (Budap).

[20] Bhargava M, Kumar V, Pandey H, Singh V, Misra V, Gupta P (2020). Role of Hematological Indices as a Screening Tool of Beta Thalassemia Trait in Eastern Uttar Pradesh: An Institutional Study. Indian J Hematol Blood Transfus.

[21] Bordbar E, Taghipour M, Zucconi B E (2015). Reliability of Different RBC Indices and Formulas in Discriminating between β-Thalassemia Minor and other Microcytic Hypochromic Cases. Mediterr J Hematol Infect Dis.

[22] Cousens N E, Gaff C L, Metcalfe S A, Delatycki M B (2010). Carrier screening for beta-thalassaemia: A review of international practice. Eur J Hum Genet.

[23] Mentzer W C (1973). Differentiation of iron deficiency from thalassemia trait. Lancet.

[24] Shine I, Lal S (1977). A strategy to detect beta-thalassemia minor. Lancet.

[25] England J M, Fraser P M (1973). Differtentiation of iron deficiency from thalassemia trait by routine blood count. Lancet.

[26] Green R, King R (1989). A new red cell discriminant incorporating volume dispersion for differentiating iron deficiency anemia from thalassemia minor. Blood Cells.

[27] Srivastava P C, Bevington J M (1973). Iron deficiency and/or thalassemia trait. Lancet.

[28] Ricerca B M, Storti S, D'onofrio G, Mancini S, Vittori M, Campisi S, Mango G, Bizzi B (1987). Differentiation of iron deficiency from thalassemia trait: A new approach. Haematologia (Budap).

[29] Sirdah M, Tarazi I, Al N E, Al H R (2007). Evaluation of the diagnostic reliability of different RBC indices and formulas in the differentiation of the beta-thalassaemia minor from iron deficiency in Palestinian population. Int J Lab Hematol.

[30] Ehsani M, Darvish A, Aslani A, Seighali F (2005). A new formula for differentiation of iron deficiency anemia (IDA) and thalassemia trait (TT). Turk J Haematol.

[31] Telmissani O A, Khalil S, George T R (1999). Mean density of hemoglobin per liter of blood: A new hematologic parameter with an inherent discriminant function. Lab Hematol.

[32] Nishad A A N, Pathmeswaran A, Wickramasinghe A R, Premawardhena A (2012). The Thal-index with the BTT prediction. exe to discriminate β-thalassaemia traits from other microcytic anaemias. Thalassemia Reports.

[33] Wongprachum K, Sanchaisuriya K, Sanchaisuriya P, Siridamrongvattana S, Manpeun S, Schlep F P (2012). Proxy indicators for identifying iron deficiency among anemic vegetarians in an area prevalent for thalassemia and hemoglobinopathies. Acta Haematol.

[34] Pornprasert S, Panya A, Punyamung M, Yanola J, Kong-Pan C (2014). Red cell indices and formulas used in differentiation of β-thalassemia trait from iron deficiency in Thai school children. Hemoglobin.

[35] Sirachainan N, Iamsirirak P, Charoenkwan P, Kadegasem P, Wongwerawattanakoon P, Sasanakul W, Chansatitporn N, Chuansumrit A (2014). New mathematical formula for differentiating thalassemia trait and iron deficiency anemia in thalassemia prevalent area: A study in healthy school-age children. Southeast Asian J Trop Med Public Health.

[36] Sehgal K, Mansukhani P, Dadu T, Irani M, Khodaiji S (2015). Sehgal index: A new index and its comparison with other complete blood count-based indices for screening of beta thalassemia trait in a tertiary care hospital. Indian J Pathol Microbiol.

[37] Matos J F, Dusse L M, Borges K B, de Castro R L, Coura-Vital W, Carvalho M (2016). A new index to discriminate between iron deficiency anemia and thalassemia trait. Rev Bras Hematol Hemoter.

[38] Jahangiri M, Rahim F, Malehi A S (2019). Diagnostic performance of hematological discrimination indices to discriminate between beta thalassemia trait and iron deficiency anemia and using cluster analysis: Introducing two new indices tested in Iranian population. Sci Rep.

